# Antibody-Drug Conjugates for Cancer Therapy: Chemistry to Clinical Implications

**DOI:** 10.3390/ph11020032

**Published:** 2018-04-09

**Authors:** Nirnoy Dan, Saini Setua, Vivek K. Kashyap, Sheema Khan, Meena Jaggi, Murali M. Yallapu, Subhash C. Chauhan

**Affiliations:** Department of Pharmaceutical Sciences and Cancer Research Center, University of Tennessee Health Science Center, Memphis, TN 38163, USA; ndan@uthsc.edu (N.D.); ssetua@uthsc.edu (S.S.); vkashya1@uthsc.edu (V.K.K.); skhan14@uthsc.edu (S.K.); mjaggi@uthsc.edu (M.J.)

**Keywords:** antibody, drug conjugation, chemical linker, drug delivery, and cancer therapy

## Abstract

Chemotherapy is one of the major therapeutic options for cancer treatment. Chemotherapy is often associated with a low therapeutic window due to its poor specificity towards tumor cells/tissues. Antibody-drug conjugate (ADC) technology may provide a potentially new therapeutic solution for cancer treatment. ADC technology uses an antibody-mediated delivery of cytotoxic drugs to the tumors in a targeted manner, while sparing normal cells. Such a targeted approach can improve the tumor-to-normal tissue selectivity and specificity in chemotherapy. Considering its importance in cancer treatment, we aim to review recent efforts for the design and development of ADCs. ADCs are mainly composed of an antibody, a cytotoxic payload, and a linker, which can offer selectivity against tumors, anti-cancer activity, and stability in systemic circulation. Therefore, we have reviewed recent updates and principal considerations behind ADC designs, which are not only based on the identification of target antigen, cytotoxic drug, and linker, but also on the drug-linker chemistry and conjugation site at the antibody. Our review focuses on site-specific conjugation methods for producing homogenous ADCs with constant drug-antibody ratio (DAR) in order to tackle several drawbacks that exists in conventional conjugation methods.

## 1. Introduction

Cancer, responsible for about 8.2 million deaths per year globally, is the second most common deadly disease which severely affects the human health worldwide [1]. Current treatment options for cancer include surgery, chemotherapy, radiation, and immunotherapy. Modern approaches to combat cancer include stem cell therapy, hyperthermia, photodynamic therapy, laser treatment, etc. Among these therapeutic interventions, chemotherapy, either alone or in combination with surgery or radiation therapy, is the most widely used therapeutic option. Neoadjuvant chemotherapy is used to shrink tumors before surgery or radiation. Adjuvant chemotherapy is employed after surgery or radiation to kill remaining cancer cells. Conventional chemotherapy is often associated with a low therapeutic window due to poor pharmacokinetic properties of the drugs used. Additionally, chemotherapeutic agents are not specific to tumor cells and they can affect normal cells with high mitotic rates. This can lead to life-threatening side effects in cancer patients. The severity of such uninvited side effects can be reduced by conjugating different types of highly potent un-targeted drugs such as tubulin polymerization inhibitors, DNA damaging agents conjugated to a monoclonal antibody (mAb). Another proven approach to minimize chemotherapy side effects is nanotechnology. This approach gives the ability to load drug molecules in polymer/metal nanoparticles, liposomes, micelles, self-assemblies, and nanogels. Nanotechnolgy enhances passive delivery of chemotherapeutics to malignant cells (due to leaky vasculature) [2]. However, the existence of abundant stromal/fibrosis (desmoplasia) in tumors, leads to inefficient drug delivery and emergence of drug resistance [3]. All these unmet needs imply the importance to consider alternative approaches for successful therapeutic intervention in cancer. 

**Figure 1:**
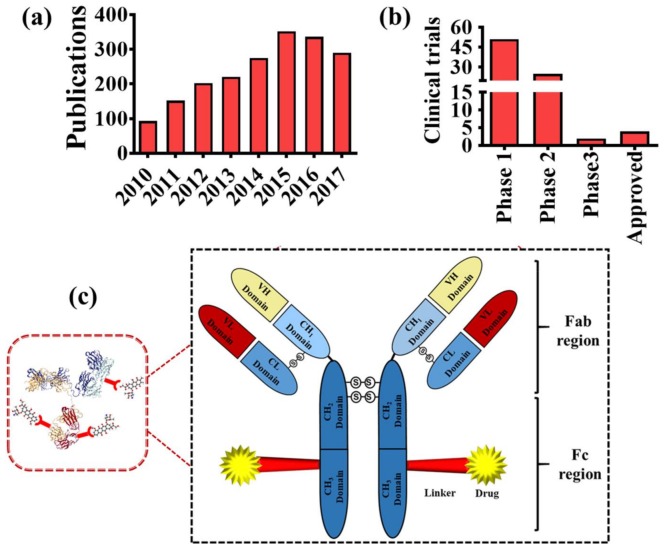
(**a**) Yearly peer-reviewed articles on ADCs based on PubMed search; (**b**) Registered clinical trials of ADCs based on Clinicaltrials.gov database; (**c**) Key components of an ADC.

A site-specific targeted delivery of cytotoxic drugs is proving to be a better option for efficient drug delivery. This can be achieved by conjugating cytotoxic drugs to a suitable and validated mAb. ADC strategy not only enhances the therapeutic window of potent cytotoxic drugs, but also minimizes chemo-associated side effects. ADCs attain the idea of a “magic bullet” conceptualized by Paul Ehrlich [4]. The concept of ADC in drug development was well recognized following the Food and Drug Administration (FDA) approval for Adcetris^®^ (brentuximab vedotin) in 2011 and Kadcyla^®^ (trastuzumab emtansine) in 2013. These successes prompted enormous interest among antibody guided therapeutic researchers from both academia and industry. This is evident from a sharp increase in related publication in PubMed (Figure 1a) and registered clinical trials in different phases of various types of ADCs (Figure 1b).

**Figure 2:**
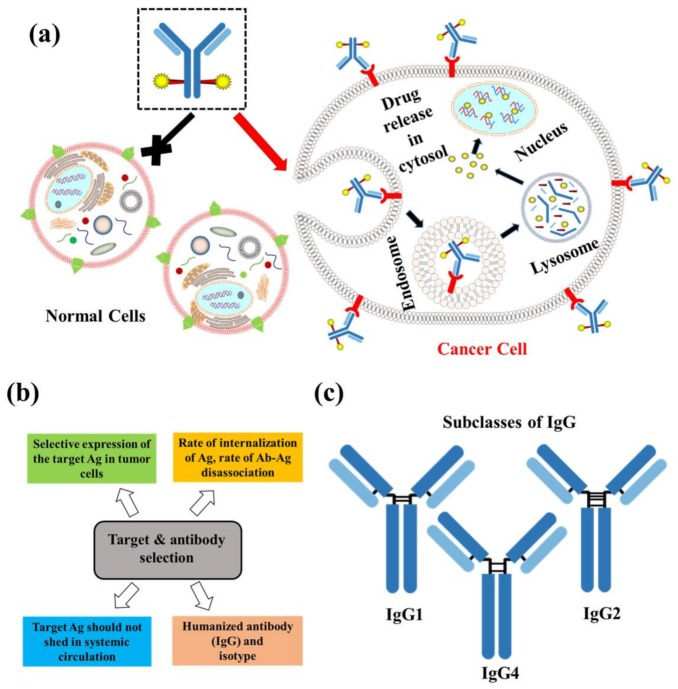
(**a**) Schematic representation of ADC uptake in cells expressing target antigen followed by release of the payload; (**b**) Key considerations while choosing target and antibody isotype for ADC developments; and (**c**) subclasses of IgG.

ADCs are typically comprised of a fully humanized mAb targeting an antigen specifically/preferentially expressed on tumor cells, a cytotoxic payload, and a suitable linker (Figure 1c). This composition mainstay preserves cytotoxicity of drugs, targeting characteristics, and stability of ADCs in systemic circulation. The right combination of selection is key in developing a succeessful ADC. To acheive specific delivery of a cytotoxic payload, the target antigen must be highly expressed on the surface of the tumor cells rather than the normal cells. Conjugating a mAb to highly potent cytotoxic payloads facilitates site-specific delivery of the payload to the target cells, thus minimizing the chances of off target cytotoxicity. Upon binding to the specific antigen, the antibody gets absorbed through rapid internalization followed by lysosomal degradation, and subsequently releasing the cytotoxic drug inside the cell (Figure 2a). This way, ADCs can be used to deliver cytotoxic drugs to cancer cells [5]. 

Over the past 30 years of ADC research, several new linkers, and conjugation strategies have been discovered. However, very few of them have reached to the clinic. This shows the degree of difficulty in optimizing key parameters of ADC such as choosing a potent cytotoxic payload, a suitable target, a stable linker, the conjugation site to the mAb, and the conjugation technology. Therefore, in this review, we attempt to discuss various advancements and challenges in ADC technologies with a special focus on linkers and conjugation methods. 

## 2. Composition of ADCs

### 2.1. Target and Antibody

The conjugation of potent cytotoxic drug molecules to mAbs demonstrates a promising approach for the development of targeted cancer therapy. The selection of mAbs for specific targeting and harnessing the therapeutic drug molecule(s) with mAbs represents ADCs precision acting only on cancer cells, increasing the therapeutic index while minimizing the off-target effects (Figure 2b). Therefore, determining which antigen to target is the first major step in ADC development. The target antigen should be overexpressed on tumor cells surface homogenously with relatively low to no expression on healthy cells to ensure site-specific targeting delivery of cytotoxic payloads [6]. Immunohistochemistry, flow cytometry, tissue microarrays, reverse transcription polymerase chain reaction (RT-PCR), messenger RNA (mRNA) profiling are commonly used to anlyze tumor expression of the target antigen in patient tissue samples [7]. Upon confirming antigen overexpression, mAbs against this particular antigen are generated through Hybridoma Technology (a method for producing monoclonal antibodies). The hybridoma cells are immortalized by fusing antibody producing B cells from mice and mouse myeloma cells, then they are further cultured to generate monoclonal antibodies of interest [8]. Selection of mAbs for generation of ADCs is based on their tumor penetrating ability and binding affinity (Kd < 10 nM) [5]. MAbs with strong binding on antigens were found to be confined in perivascular spaces, whereas as low binding affinity mAbs can internalize well inside the tumor [9]. Thus, a balance between internaliztion and disassociation rates of the antigen-antibody (Ab-Ag) complexes governs effective delivery of the payload to the tumor space. Sometimes shedding of a target antigen from tumor tissues or the presence of a circulating antigen in systemic circulation, in a considerable amount, can alter the potency and pharmacokinetics of the ADCs. In these circumstances a significant amount of the payload on ADCs will be lost in systemic circulation and cleared by the liver [10,11]. Studies on drug conjugates with shedding antigens, like tumor-associated carbohydrate antigen CanAg (a glycoform of mucin 1) and carcinoma antigen 125 (CA-125), showed no decrease in therapeutic efficacy [11,12]. The mAbs used for drug cross-linking are of the IgG isotype (Figure 2c) specifically IgG1, because of their inherent ability to trigger immune-mediated effector functions. This includes antibody-dependent cellular cytoxicity (ADCC) and complement-dependent cytotoxicity (CDC) by binding to Fcγ receptors and complement C1q protein complex, respectively [13]. Independent functions of mAbs can be an added advantage over the cell killing potency of the ADC warhead but they can contribute to toxicity sometimes [14]. For instance, anti-HER2 trastuzumab, in trastuzumab-emtansine (DM1) contributes to the antitumor efficacy of the ADC by mediating antibody-dependent cell-mediated cytotoxicity (ADCC) [15]. IgG2 and IgG4 isotypes can be used in ADCs but are less efficient in modulating effector functions and delivery in comparison to IgG1 [7,13]. IgG3 isotypes have a lower half-life, exteneded hinge region compraed to other isotypes and they are prone to polymorphisms and immunogenic reactions. Immunogenicity caused by previouly used murine and chimeric mAbs is countered by converting them to humanized mAbs. In humanized mAbs, the Fc region is from the human source and complementarity determining region are from non-human (rat/mouse) sources [16].

### 2.2. Linker

**Figure 3:**
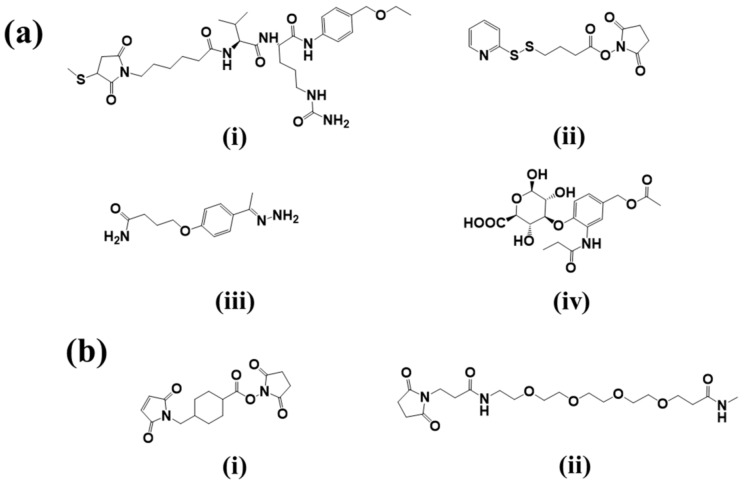
Chemical structures of linkers used in ADCs development. (**a**) Key cleavable linkers: (i) Lysosomal protease sensitive Val-Cit dipeptide linker; (ii) Glutathione sensitive SPDB linker; (iii) Acid Sensitive AcBut linker; and (iv) β-Glucuronidase sensitive linker; and (**b**) non-cleavable linkers: (i) SMCC linker; and (ii) PEG4Mal linkers.

One of main challenges in developing ADCs is to incorporate a linker that will maintain the stability of the ADC in systemic circulation for a prolonged period and release the payload after internalization at the target site. The site of conjugation and choice of linker play a critical role in the stability, the pharmacokinetic properties of ADCs. Attachment sites in antibody mAb can also be engineered via several ways for incorporation of a linker and subsequently the drug. Based on release mechanism, linkers are generally divided as cleavable (Figure 3a) and non-cleavable linkers (Figure 3b).

#### 2.2.1. Cleavable Linkers

- *Acid-sensitive linkers*: Acid-sensitive hydrazone groups in acid-labile linkers remains stable in systemic circulation (pH 7.5) and gets hydrolyzed in lysosomal (pH 4.8) and endosomal (pH 5–6) acidic tumor micro-environment upon internalization in the targeted cells [17]. Withdrawal of gemtuzumab ozogamicin (Mylotarg^®^) in 2010, an anti-CD33 ADC for treatment of acute myeloid lymphoma, raises concern over the stability of this linker [18]. The heterogeneous nature of the drug conjugate contributed to premature release of payload, which in turn may have contributed to its remarkable toxicity compared to conventional chemotherapy. Currently, inotuzumab ozogamicin and milatuzumab doxorubicin, that are developed with a hydrazone linker.
- *Glutathione-sensitive disulfide linkers*: Another common example of cleavable linkers is glutathione-sensitive disulfide linkers. Glutathione is a low molecular weight thiol which is present in the cytoplasm (0.5–10 mmol/L) and extracellular environment (2–20 µmol/L in plasma) [19]. In tumor cells elevated levels of thiols are found during stress conditions such as hypoxia [20]. The difference in glutathione concentration in cytoplasm and extracellular environment can be implemented as a selective delivery of the drug payload to target tumor via breakdown of disulfide linkers [21]. Besides glutathione, intercellular protein disulfide isomerase (PDI) is also capable to reduce disulfide bonds. Two cysteine residues in the active site of this enzyme governs the thiol-disulfide exchange reactions with or within substrates [22]. Maytansinoid drug conjugates have been widely employed for disulfide bonds with an average DAR of 3–4 [23].
- *Lysosomal protease-sensitive peptide linkers*: Tumor cells have higher expression of lysosomal proteases like cathepsin B than normal cells. Cathepsin B-sensitive peptide linker conjugated ADCs selectively binds to and get internalized into tumor cells via receptor mediated endocytosis [24]. Proteases are inactivated in serum in presence of a high pH and different serum protease inhibitors [24]. This makes the peptide linker stable in systemic circulation and only to be cleaved upon internalization in tumors. In case of the FDA approved Adecetris^®^, cathepsin B- sensitive valine-citruline linker is found to be superior to hydrazone linker. The valine-citruline linker connects the bridge between *p*-aminobenzylcarbamate-monomethyl auristatin E (MMAE) and anti-CD30-mAb [5].
- *β-glucuronide linker*: β-Glucuronidase-sensitive linkers have been successfully used in a handful of glucuronide prodrugs [25]. Lysosomes and tumor necrotic areas are rich in β-glucuronidase which is active at lysosomal pH and inactive at physiological pH [26]. This selective site of action allows for a selective release of cytotoxic payloads through cleavage of the glycosidic bond of β-glucuronidase-sensitive β-glucuronide linkers. Further, the hydrophilic nature of this linker provides aqueous solubility for hydrophobic payloads and decreases aggregation of ADCs [27]. A highly hydrophobic CBI payload was conjugated to h1F6 and cAC10 mAbs utilizing β-glucuronide linker with an average DAR ~4–5 [27]. Such ADC compositions were found to be mostly monomeric in nature compared to extremely aggregated PABC-dipeptide based CBI conjugates [27]. Psymberin/irciniastatin A, a phenolic cytotoxic payload-based ADC was developed with *N*,*N*′-dimethylethylene diamine self-immolative spacers and a β-glucuronide linker for targeting CD-30-positive and CD-70-positive malignancies [28]. This development led to the possibility of developing phenolic warhead-based ADCs as many anti-cancer drugs have phenol functional groups. Another β-glucuronidase-sensitive linker based ADC has recently been developed utilizing tertiary amine functional group of payloads (tubulysins and auristatin E) as the conjugation site to the linker [29]. Tertiary-ammonium based linkers provide an excellent strategy for conjugating payloads without affecting their activity [29].

#### 2.2.2. Non-Cleavable Linkers

ADCs with non-cleavable thioether linkers have better plasma stability. Higher plasma stability decreases the non-specific drug release of ADCs as compared to cleavable linkers [30]. The linker is attached to the amino acid residues of the mAb through a nonreducible bond, accounting for high plasma stability. Following internalization, the drug is released from these conjugates due to lysosomal proteolytic degradation of the mAb. The drug-linker-amino acid residue itself must retain the activity of the drug [31]. FDA approved trastuzumab emtansine (Kadcyla^®^/T-DM1) uses a non-cleavable SMCC (*N*-succinimidyl-4-(maleimidomethyl) cyclohexane-1-carboxylate) linker to crosslink the warhead DM1 to lysine residues of anti-HER2 mAb trastuzumab. The intercellular metabolite lysine-MCC-DM1 complex was found to be as active as the parent drug, DM1, after lysosomal degradation of trastuzumab [32]. The hydrophobic nature of lysine-MCC-DM1 metabolites restricted the bystander effect and caused aggregation leading to immunogenicity. Polarity of the DM1 conjugates was increased with a tetramer PEG4Mal linker. Lysine-PEGMal-DM1 metabolites were found to be more potent, effectively retaining in MDR1 expressing cancer cells compared to lysine-MCC-DM1 metabolites [33]. Monomethyl auristatin F (MMAF) conjugates with non-reducible thioether linker were found to be highly stable with equal potency as compared to valine-citrulline conjugates [34].

#### 2.2.3. Rational Linker Design to Overcome Resistance

Increasing occurrence of resistance in cancer patients, is a major challenge in anti-cancer drug discovery. Resistance to ADCs can be inherent or acquired and can be caused by several reasons including overexpression of efflux transporter proteins, downregulation or altered expression of target antigen, activation of different signaling pathways, blocked binding site at the target antigen etc. [35]. Commonly used cytotoxic payloads in ADC, such as calicheamicin, auristatins, maytansines, taxnes, and doxorubicin are well-known substrates of efflux transporter of P-gp, which pumps out the drug from intracellular space. This commonly observed phenomenon is key to the development of multi drug resistance (MDR), where patients fail to respond to several chemotherapies [36]. Clinically approved gemtuzumab ozogamicin was found to be less effective in acute myeloid leukemia patients with high expression of MDR proteins [37]. However, effective linker design can help to overcome multidrug resistance. Hydrophobic compounds are found to be more sensitive towards MDR1 efflux transporter. In a study with the hydrophilic DM1 payload, incorporation of a hydrophobic PEG4Mal linker enhanced potency of the ADC in MDR1 containing xenograft models [33]. When compared to SMCC linker, lysine-PEG4Mal-DM1 metabolites were more accumulated in MDR expressing COLO 205 cells than lysine-SMCC-DM1 metabolites [33]. In cleavable linkers, the payload gets more sensitive after it is released from the linker whereas, payloads with non-cleavable linkers are found to be less susceptible towards efflux proteins, where the mAb is digested but the linker-payload metabolite remains active [38]. Further, Zhao and co-workers showed that incorporation of a non-charged PEG group or negative charged α-sulphonic acid group increased hydrophilicity of commonly available hydrophobic SPDP, SMCC linkers as well as provided better therapeutic window for maytansine conjugates against MDR1 expressing cell lines in vitro and in vivo [39]. 

### 2.3. Payloads

**Figure 4:**
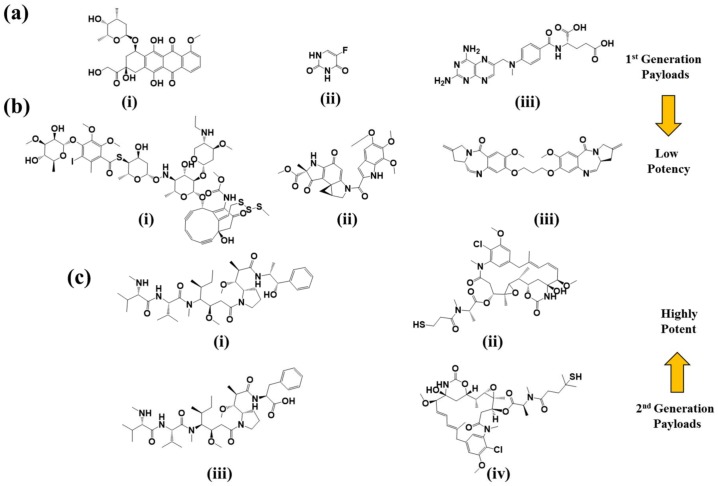
Chemical structures of first and second generation payloads used in ADCs. (**a**) 1st generation ADC payloads: (i) doxorubicin; (ii) 5-fluorouracil; and (iii) methotrexate; (**b**) DNA damaging agents: (i) calicheamicin γ1; (ii) duocarmycin A; and (iii) SJG-136 PDB dimer; and (**c**) tubulin polymerization inhibitors: (i) monomethyl auristatin E (MMAE); (ii) mertansine (DM1), monomethylauristatin F (MMAF), and ravtansine (DM4).

Clinically approved chemotherapeutics with known clinical profiles like doxorubicin, methotrexate, and 5-flurouracil (Figure 4a) were commonly used as payloads in ADCs [40]. 

The clinically approved topoisomerase II inhibitor doxorubicin conjugated is to the hinge region cysteine residues of BR96 humanized mAb through an acid sensitive hydrazone linker is one of the 1st generation ADCs targeting the Lewis Y antigen, found to be overexpressed in different cancers [41]. The effective dose of BR96-doxorubicin in in vivo studies was found to be very high due to low potency of the payload (IC_50_ 0.1–0.2 µM) although with a high DAR value of 8. Despite such promising preclinical data, the BR96-doxorubicin conjugates failed to show enough efficacy in clinical trials, while patients experienced significant gastrointestinal toxicity due to the presence of Lewis Y in the gut [42]. Lessons from the story of BR96-doxorubicin established the use of more potent cytotoxic payloads, preferably with IC_50_ values in the sub-nanomolar range, as there is only a certain amount of payload that can be delivered via ADCs. Antibody conjugated delivery of highly potent chemotherapeutic drugs can increase tumor specificity, therapeutic index as well as decrease systemic toxicity. The cytotoxic payloads used in ADCs development can be divided in two classes based on their mechanism of action, DNA damaging agents (Figure 4b) and tubulin inhibitors (Figure 4c).

#### 2.3.1. DNA Damaging Agents

Calicheamicins are naturally occurring highly potent DNA damaging agents isolated from the fermentation broth of a soil microorganism *Micromonospora echinospora* ssp. *Calichensis* [43]. Upon binding at the minor grove of the DNA they are reduced by cellular thiols to form a 1,4-dehydrobenzene radical intermediate, which then removes hydrogen from the deoxyribose ring and breaks the DNA strand [44] through a reaction commonly known as Bergman cyclization [45]. Calicheamicin was found to alter the expression of different key cell elements at the transcriptional level such as ribosomal proteins, nuclear proteins, and proteins accountable for stress response, different genes involved in DNA repair/synthesis, as well as metabolic and biosynthetic genes [46]. Calicheamicin is being investigated as payload in several ADCs; gemtuzumab ozogamicin and inotuzumab ozogamicin are noteworthy among them. Gemtuzumab ozogamicin incorporates a hydrazide derivative of calicheamicin, *N*-acetyl-γ-calicheamicin dimethyl hydrazide. CD-22 selective inotuzumab ozogamicin also incorporates *N*-acetyl-γ-calicheamicin dimethyl hydrazide as a payload [47]. Duocarmycins and pyrrolobenzodiazepines (PBD) are other notable chemotherapeutics in this class which are now in early stages of clinical development for payloads in ADCs [44]. Duocarmycins (DNA minor groove binders) exerts their cytotoxicity by alkylating adenine residues at the N3 position of DNA strands. SYD983, a duocarmycin-trastuzumab ADC was recently developed [48]. Of note, potent PBDs are naturally produced by *actinomycetes*. They covalently bind to a particular sequence in DNA minor groove and form an amine bond in-between C11 of PBD and N2 of guanine bases [49]. Although they do not disrupt the DNA structure considerably, formation of DNA-PBD adduct impedes key DNA functions like transcription and translation [50]. Several ADCs currently under clinical trial (SGN-CD70A, SGN-CD 33A and SGN-CD123A from Seattle Genetics) employ a PBD dimer SGD-1882 as their payload [51]. ADCs with PDB dimers are also found to be involved in bystander killing [50].

#### 2.3.2. Tubulin Polymerization Inhibitors

Tubulin polymerization inhibitors (auristatins and maytansinoids) are widely employed as cytotoxic payloads [44]. Auristatins are water-soluble synthetic analogs of a marine natural product (dolastatin 10) isolated form the extract of a sea hare, *Dolabella auricularia*. The parent compound was also found in cyanobacteria *Symploca hydnoides* and *Lyngbya majuscula*, which are nourishment to the sea hare [52,53]. Dolastatin 10, a series of linear peptides comprised of dolavaline, valine, dolaisoleuine, dolaproine amino acid residues and a complex primary amine (dolaphenine) is found to be active against a wide range of cancer cell lines and solid tumors at very low concentrations (average IC_50_ value is in sub nanomolar range) [52,54]. It shares the same tubulin-binding site as vinca alkaloids and inhibits tubulin polymerization and tubulin-dependent GTP hydrolysis that causes cell cycle arrest in the G2/M phase, eventually leading to cell death [55]. Seattle Genetics has developed two auristatin derivatives (MMAE and MMAF), which are currently being used as payloads in several ADCs by linking to the cysteine residues of the mAb [56,57,58]. Bentuximab vedotin, a FDA approved ADC, incorporates MMAE which is linked to the cysteine residues of anti-CD30 antibody by a protease sensitive valine-citrulline dipeptide linker with an average 4 drug molecules per antibody [56]. Bentuximab vedotin is taken up in-to cytosol via cell-mediated endocytosis, where the linker is selectively cleaved in the presence of elevated lysosomal protease cathepsin B [59]. MMAE can penetrate the cell membrane, and as a result it can prompt bystander killing where it diffuses through nearby cells independent of antigen expression; by contrast, MMAF is impermeable to cell membrane [60]. This is because MMAF is more hydrophilic, less potent, and less toxic than MMAE. The presence of a charged phenylalanine moiety at the C-terminus of MMAF structure perturbs its cell membrane permeability [34]. Maytansiniod derivatives DM1 and DM4 are another type of microtubule polymerization inhibitors that are developed by Immunogen. Maytansine, an ansa antimitotic isolated from the bark of Ethiopian shrubs *Maytenus ovatus* and *Maytenus serrata* shares the same tubulin binding site and mechanism of action as vinca alkaloids and destabilizes microtubule assembly resulting cell cycle arrest in G2/M phase [61,62,63]. DM1 and DM4 are maytansinoids with methyl disulfide substitutions at the C3 *N*-acyl-*N*-methyl-l-alanyl ester side chain of maytansine [64]. Clinically approved Kadcyla^®^ uses DM1 as a payload with an average DAR of 3.5 for treatment of HER2+ metastatic breast cancers [32]. SAR3419, a CD-19 targeted ADC with DM4 payload is in phase II clinical trial for the treatment of B-cell malignancies. DM4 is linked to the lysine residues of the mAbs with a thiol sensitive *N*-succinimidyl-4-(2-pyridyldithio) butyrate (SPDB) linker yielding an average DAR of 3.5 [65].

α-Amanitin, a RNA polymerase II inhibitor, is a highly water-soluble mushroom derived octapeptide, which is currently being investigated as a payload in pre-clinical ADCs [66]. In proof of concept studies α-amanitin was efficiently delivered to the target cells through an anti-HER2 mAb and the IC_50_ values were found to be in pico molar range [67]. An anti-EpCAM ADC conjugated with α-Amanitin payload via a protease/esterase sensitive glutamate linker was also found to be highly effective in EpCAM expressing tumor models [68]. Recently, anti-PSMA-α-amanitin ADCs were successfully employed to reduce tumor growth in preclinical prostate cancer model with stable and cleavable linker [69]. 

## 3. Conjugation

Mylotarg^™^ was the first ADC to be approved by the FDA, first marketed in 2000 until it’s voluntary withdrawal in 2010 due to lack of significantly improved clinical benefits. The heterogeneous nature of drug conjugates and hydrazine linker instability were thought be accountable for the failure of this ADC. Thus, there was an urgent need for developing new strategies for producing homogenous drug antibody conjugation methods. Several strategies have been employed for cross-linking the antibody to drug by a linker using solvent reachable reactive amino acids with nucleophilic groups in antibody side chains. Side chain cysteine (SH group) and lysine (NH_2_ group) have been extensively used for conjugation (Table 1). The main problem with these conventional conjugation methods is the heterogeneous nature of the end products with different DAR values [70]. The conjugation strategy must not alter any key blocks of an antibody that are responsible for its binding to the target antigens. 

### 3.1. Via Side Chain Cystine Residues

Conjugation via side chain cysteines is a widely utilized and accepted technology in conjugation chemistry of ADCs. Seattle Genetics’ ADC brentuximab vedotin utilizes this method to conjugate MMAE with the anti-CD30 mAb (cAC10) via an enzymatically cleavable dipeptide linker [71]. Cysteines are engaged in interchain and intrachain disulfide bridges in an antibody, which did not contribute to the building blocks of an antibody. In an IgG1 antibody, there are four interchain disulfide bonds [72,73]. It was also found that interchain disulfide bonds are more susceptible to reduction than intrachain disulfide bonds, which allow for a controlled reduction of the four interchain disulfide bonds with dithiothreitol (DTT) or tris(2-carboxyethyl)phosphine (TCEP) while keeping intrachain disulfide bond intact. This can yield up to eight reactive sulfhydryl groups, facilitating drug conjugation with DAR values of 0–8 [70,74]. These reactive sulfhydryl groups which are nucleophilic in nature, can be reacted with electrophiles like maleimides, haloacetyls for crosslinking proteins [75]. Conjugation via cysteine produces more uniform products than lysine conjugation that are easier to purify and characterize pharmacokinetically. Previously mentioned problems with non-specific conjugation methods established a need for more specific methods for conjugation.

### 3.2. Via Side Chain Lysine Residues

Mylotarg^™^ had utilized side-chain reactive lysine residues of a humanized anti-CD33 mAb for conjugating the drug calicheamicin by a bifunctional acid sensitive hydrazone linker [76]. However, Pfizer voluntarily withdrew this product in 2010 [77]. Ado-trastuzumab-emtansine (Kadcyla^®^), one of four approved ADCs in the market utilizing side chain lysines for conjugating the potent tubulin inhibitor emtansine to mAb trastuzumab (Herceptin^®^) [32]. An ESI-TOF MS method confirms that 40 out of 86 lysine residues of humanized monoclonal IgG1 huN901-antibody are available for conjugation to DM1 molecules. Peptide mapping further showed conjugation sites present in both the heavy and light chain [78]. 

### 3.3. Drug Antibody Ratio (DAR)

DAR is defined as the number of drug molecules per mAb. DAR plays a definitive role in developing ADCs, as it determines the dose needed to produce the desired effect in patients. There is a limited number of drug molecules that can be efficiently delivered to the target site and drug loading significantly contributes to the pharmacokinetic profile of ADC. Hamblett and co-workers showed that the effect of drug distribution on the different properties like therapeutic window, pharmacokinetic properties, and maximum tolerated dose of cAC10-MMAE conjugates. Decreasing the DAR resulted in a superior therapeutic window of cAC10-MMAE conjugates, proving that drug loading as a decisive parameter for designing ADCs. Although cAC10-MMAE conjugates with DAR ~2–4 were less active in in vitro studies, but their results in in vivo studies were found to be equivalently potent (DAR~4) and better tolerated than the conjugate with higher DAR ~8. Similar observations were found with regards to pharmacokinetic properties [70]. If fewer drug molecules are conjugated per mAb, the ADC system will not be effective clinically. On the other hand, conjugating too many drug molecules per mAb will make the ADC unstable, toxic and may lead to aggregation and immunogenic reactions [79,80]. Hydrophobic MMAE conjugates using interchain cysteines with higher DAR are found to be physically unstable [79]. Normally ADCs contain different species with differing DAR values and every species has its own distinct pharmacokinetics. ADCs with heavily loaded drugs are more rapidly cleared from the system. In general, an average DAR of 3–4 is used to achieve optimum effect in ADCs, depending upon potency of the payload [70,81]. However, a recently developed poly-1-hydroxymethylethylene hydroxymethylformal (PHF) polymer-based ADC with a higher DAR of ~20 challenged this conventional concept. With vinca alkaloid as the payload and trastuzumab as the targeting mAb, the newly developed platform not only showed promising activity in xenograft tumor models, but also demonstrated good pharmacokinetic properties [82]. Conjugations through side-chain lysine residues are highly heterogeneous leading to inconsistent DAR values and different conjugation sites in the antibody. In case of Kadcyla^®^ where the drug DM1 was conjugated with the trastuzumab through the side chain lysine residues, an average DAR was found to be ~3.5 [83]. Side chain cysteine conjugation employs a controlled reduction of four intrachain disulfide bonds that allows conjugation of 0–8 drug molecules per antibody [84]. Common analytical methods for determining DAR are UV-Vis spectroscopy, hydrophobic interaction chromatography (HIC), LC-ESI-MS and rpHPLC. UV visible spectroscopy exploits the dissimilarities in maximum wave length absorbance of payload and mAb for determining respective concentrations [85]. UV-Vis spectroscopic method is widely employed to characterize huN901-DM1, 791T/36-methotrexate and cAC10-MMAE conjugates [70,86,87]. HIC uses a column consisting of a hydrophobic stationary phase and a mobile phase with gradient salt concentration to separate ADC species based on hydrophobic interactions. Mostly the ADC payloads are hydrophobic in nature, and hydrophobic conjugated species are retained in the column, whereas unconjugated species elute first in neutral pH and non-denaturizing conditions [88]. This method is more compatible with ADCs with cysteine conjugation sites on mAb, while LC-ESI-MS method was developed for characterizing lysine-conjugated ADCs [89,90]. LC-MS is advantageous over HIC or UV-Vis spectroscopic characterization as it not only gives information on DAR or drug distribution but also gives crucial structural insights of ADCs at the molecular level [91]. Wagner-Rousset and co-workers designed a simple and fast method of DAR determination based on antibody-fluorophore conjugates (AFCs) with the same linker and conjugation chemistry as ADCs. Instead of toxic payloads, a non-toxic dansyl sulfonamide ethyl amine payload was used. AFCs were subjected to digestion by *Streptococcus pyogenes* (IdeS) accompanied by DTT reduction, which generated seven easily ionizable fragments (Fd0, Fd1, Fd2, Fd3. L0, L1, Fc/2) of ~25 kDa. These resultant fragments were analyzed by LC-ESI-TOF-MS method. This method is advantageous over single step reduction as it not only gives routine information like DAR and drug distribution but also provides crucial structural details like *N*-glycosylation profiling, C-terminal lysine truncation, pyroglutamylation, oxidation and degradation products [92].

### 3.4. Site Specific Conjugation

The most common problems with conventional conjugation technologies are heterogeneous byproducts with different drug distributions per mAb, un-conjugated and overly conjugated mAbs. These phenomena are attributed to poor pharmacokinetic properties and instability of ADCs in systemic circulation [70]. Un-conjugated antibodies occupy the site of attachment, competing with drug-conjugated antibodies and block the site for internalization for the targeting mAb. On the other hand, overly conjugated mAbs are more rapidly cleared as well as can cause immunogenic reactions and toxicity. Engineering of the conjugation site may lead to a more homogenous product with defined and uniform drug stoichiometry (Table 2).

#### 3.4.1. Engineering of Side Chain Cysteine Residues

The first site-specific conjugation method for cysteine residues was developed at Genentech [93]. In this method, potential sites, which do not contribute to pivotal functions of the mAb like antigen folding or binding were identified and mutated with cysteine residues to generate a novel platform, called Thiomab™. A phage display-based biochemical assay (Phage ELISA for Selection of Reactive Thiols, PHESELECTOR) was employed to identify tolerated reactive cysteine residues from fab region of the mAb [96]. Resultant Thiomabs are subjected to controlled reduction in presence of DTT or TCEP to produce free thiols from cysteines. Previously reduced interchain disulfides are reinstated by an oxidation process with copper sulfate or dehydroascorbic acid, while the engineered cysteines are kept in a reduced form. Thus, only the reduced cysteines were available for site-specific conjugation. In the proof of concept study, nearly homogenous (92%) anti-MUC16-Thiomab™-MC-vc-PAB-MMAE conjugates with a DAR ~2 retained activity, improved therapeutic window and were better tolerated in preclinical studies on Sprague-Dawley rats and cynomolgus monkeys when compared to conventional ADCs with higher average DAR [93]. In a different study, Thiomab™-trastuzumab-BMPEO-DM1 conjugates were also found to be better tolerated at the same dose than the conventional trastuzumab-MCC-DM1 conjugates [97]. Engineered cysteine residues at the A114C position were used for conjugation and thus site-specific conjugation at those sites lead to better linker stability, therapeutic window, and more homogenous Thiomab™ ADCs. Another site-specific conjugation approach via modifying cysteine is disulfide rebridging, where interchain disulfide bonds were reduced first and then reinstated by a bis-alkylation process to form a three carbon bridge. Resulting conjugates were found to be more stable than the maleamide conjugates in serum and high albumin concentrations [98]. 

#### 3.4.2. Incorporation of Unnatural Amino Acids (unAA)

Another powerful approach to the site-specific conjugation was developed via incorporation of unnatural amino acids (unAA) as the 21st amino acid with a reactive handle on different side chains of the mAb. It allowed selective conjugation of different classes of payloads that have not been able to be conjugated because of the limitations of conventional conjugation methods. This method also allowed conjugation ofcombination of payloads with different mechanism of action [99]. The most common method for unAA insertion employs t-RNA/amino-acyl t-RNA synthetase pair which incorporates the un-natural amino acid at the place of the amber stop codon (TAG) encoded in the gene of interest [100]. Nearly homogenous trastuzumab-MMAF conjugates with an average DAR ~2 were synthesized utilizing a site specifically introduced *p*-acetylphenylalanie unAA. The ketone group present in the *p*-acetylphenylalanie unAA formed a stable oxime linkage with the alkoxy-amine-MMAF payloads. Resulting conjugates were found to be highly stable and with a similar pharmacokinetic profile of the naked mAb [94]. In a similar study hydroxylamine-MMAD payloads conjugated to the site-specifically incorporated *p*-acetylphenylalanine residue of the 5-T4/anti-HER2 mAb via a non-cleavable linker were found to be superior to the corresponding ADCs with interchain cysteine or engineered cysteine residues as conjugation site [101].

#### 3.4.3. Enzymatic Site-Specific Conjugation Processes 

Reactive functional groups for site-specific conjugation of the drug payloads were also introduced to the antibodies by several enzymes like transglutaminase and glycotransferase. Bacterial transglutamiase from *Streptoverticillium mobaraense* forms a stable isopeptide bond in-between an amine group and g-carboxamide moiety from a glutamine tag engineered in the flexible region of the deglycosylated mAbs but not from the naturally available glutamines [95,102]. Strop and co-workers introduced a short glutamine tag LLQG into 90 different regions of an anti-EGFR antibody, among them 12 were fit for drug crosslinking. Then two (LLQGA in heavy chain and GGLLQGA in light chain) out of the 12 glutamine tags were chosen for conjugating amine containing MMAD derivatives with both the cleavable and non-cleavable linker in presence of transglutaminase. Resulting ADCs were found to be highly stable, monomeric and with an average DAR ~1.9 and better pharmacokinetic profile compared to the conventional ADCs [103]. Similar conjugates were synthesized by this method using anti-M1S1-C16 (Clone 16) mAb and an anti-Her2 mAb. A recently developed anti-Trop2 ADC, with a LLQGA glutamine tag for site-specific conjugation with an undisclosed microtubule inhibitor showed promising efficacy in preclinical studies [104]. Another additional approach for enzyme-mediated conjugation is SmartTags (Specific Modifiable Aldehyde Recombinant Tag) technology using CxPxR recognizing formyl glycine generating enzyme, which converts cysteines to formylglycine with a reactive aldehyde group [105]. Pictet−Spengler ligation chemistry allowed bio conjugation of indole based payloads to the aldehyde group of the modified mAb [106]. A modified version of Pictet-Spengler reaction is Hydrazino-Pictet-Spengler Ligation, which not only provides an effective, quick and one step conjugation as well as found to be advantageous over oxime ligation conjugation [107].

## 4. Clinical Trials

**Figure 5:**
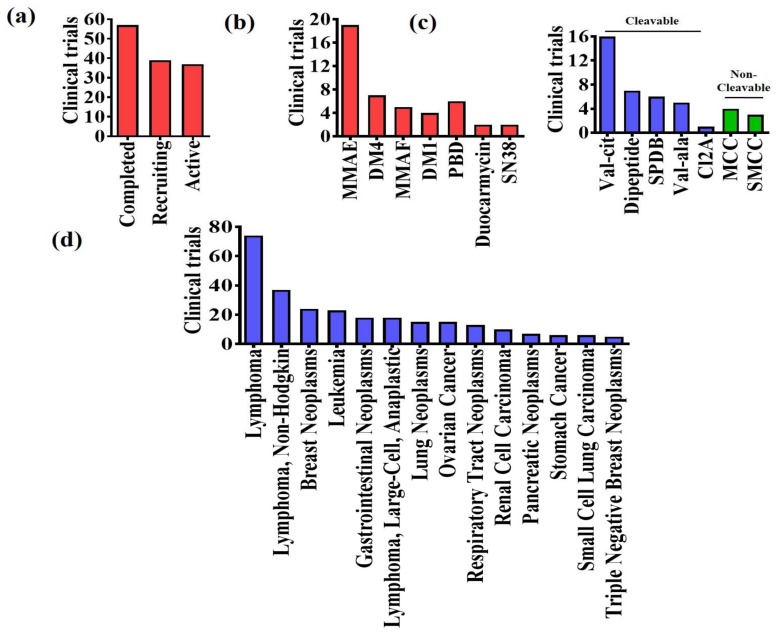
(**a**) Status of clinical trials on ADCs; (**b**) Different ADC payloads in clinical trials; (**c**) Different ADC linkers in clinical trials; (**d**) Clinical trials of ADCs for different type of oncologic indications based on clinicaltrials.gov database search.

The number of ADCs in clinical trial is rapidly increasing with two of the recently approved ADCs (Besponsa^®^, re-approved Mylotarg^™^). Currently there are more than 50 ADCs, which are in different phases of clinical trial as monotherapy as well as in combination with other chemotherapeutic drugs for treatment of different types of cancer and showing promising results. Most of the ADCs under clinical trial uses common type of payload-linker motifs although they differ in the mAb to target different types of malignancies (Figure 5). 

Among them three candidates are in phase III of clinical trials. Several ADCs are in preclinical development. In this section, we discuss about development of sacituzumab govitecan (IMMU-132), mirvetuximab soravtansine (IMGN-853), and inotuzumab ozogamicin (CMC-544), which are now investigated in phase III clinical trial.

*Sacituzumab Govitecan (IMMU-132)*: This is a moderately toxic topoisomerase I inhibitor SN38, metabolite of prodrug irinotecan conjugated to a humanized anti-Trop2 mAb by a pH sensitive CL2A linker [108]. The average DAR (7.6) of this ADC is comparatively high because of the moderately toxic payload. A short PEG spacer incorporated in-between the linker and the payload enhances the aqueous solubility of the payload. However, IMMU-132 delivers more SN-38 (active metabolite of irinotecan) to the tumor tissue than the prodrug formulation irinotecan [109]. The target trop-2 (trophoblast cell surface antigen) is over expressed in different types of cancer like breast, lung, pancreatic, colorectal, prostate and cervical [109]. Trop2 is an attractive target for triple negative breast cancer (TNBC). In phase II clinical trial, 8–10 mg/kg dose of IMMU-132 showed promising activity with manageable grade 3–4 side effects like diarrhea, neutropenia, fatigue, and anemia. No occurrence of immunogenicity was reported [110], thus a phase III trial for this drug has been initiated (NCT02574455) for refractory/relapsed TNBC patients. IMMU-132 earned Breakthrough Therapy designation from the FDA for the treatment of TNBC, small cell lung cancer, and non-small cell lung cancer. 

*Mirvetuximab Soravtansine (IMGN-853)*: It uses a humanized anti-folate receptor-α (FR-α) mAb conjugated to the maytansine payload DM-4 through a cleavable sulpho-SPDB linker. FR-α comes under class of glycoproteins that govern endocytosis mediated uptake of folates [111]. FR-α has limited expression in healthy tissues, whereas it is elevated in several malignancies [112]. A hydrophilic sulpho-SPDB linker established the bridge between the lysine residues of the mAb and the microtubule-disrupting payload DM4. After lysosomal degradation, one of the metabolites S-methyl-DM4, which is lipophilic in nature induced bystander killing in neighboring cells irrespective of the antigen expression [112,113]. Mirvetuximab soravtansine is reported to be upregulating effects of conventional chemo drugs like carboplatin in ovarian cancer [114]. In phase I dose escalation study, patients received doses from 0.15 to 7.0 mg/kg once in a three week. From the phase I study, the encouraging potency of IGMN-853 was noted for epithelial ovarian cancer with a favorable toxicity profile. From the phase I, trial results a dose of 6 mg/kg once in a three weeks was chosen for phase II clinical studies [115]. In the phase II study on patients with platinum-resistant ovarian cancer, IMGN-853 was found to be active mostly in less heavily treated individuals with a reasonable toxicity profile. Grade 2 side effects like diarrhea, nausea, blurred vision were reported [116]. Phase III clinical trial (NCT02631876) of this drug is started in patients with FR-α expressing epithelial ovarian cancer, primary peritoneal cancer or fallopian tube cancer along with a choice of chemotherapy of the investigator.

*Inotuzumab Ozogamicin (Besponsa^™^)*: Pfizer, Wyeth and University of California, Berkeley jointly developed this ADC. It entered phase III clinical trial with frontline chemotherapy in young adult patients with B Acute Lymphoblastic Leukemia (NCT03150693). It is consisted of an humanized anti-CD22 mAb G5/44 (IgG4 isotype) with a DNA damaging *N*-acetyl-γ-calicheamicin dimethyl hydrazide derivative as payload connected through an acid-sensitive 4-(4-acetylphenoxy) butanoic acid (AcBut) linker [47]. The target CD22 is a B-acute lymphoblastic leukemia (B-ALL) specific antigen with restricted expression in the surface of full-grown B cells [117]. An investigation on adult acute lymphoid leukemia patients confirmed abundance of CD22 expression [118]. Inotuzumab ozogamicin in preclinical models established it’s superiority over non-targeted conventional combination chemotherapy comprised of cyclophosphamide, vincristine and prednisone (CVP) or doxorubicin (CHOP) in in vitro studies as well as in vivo human B-cell lymphoma xenograft mice models. When it is used together with CVP, it exerted more potency but with CHOP resulted in toxicity in mice models. However, dose-dense study with 2 dosages of inotuzumab ozogamicin and CHOP found to be potent with no toxicities [119]. From the phase I study, of inotuzumab ozogamicin, MTD was found to be 1.8 mg/m^2^ with side effects like thrombocytopenia (major), asthenia, nausea and neutropenia B-cell non-hodgkin’s lymphoma [120]. Phase II clinical trial (NCT01134575) of this drug was conducted at MD Anderson Cancer Center with an adult I.V. dose 1.8 mg/m² and a pediatrics I.V. dose 1.3 mg/m². This drug was administered in 49 refractory and relapsed B-ALL patients with a median age of 36 years. The complete response rate from this study was 57% with an overall median survival rate of 7.9 months in responders [121]. Clinical trials (NCT01564784) of inotuzumab ozogamicin with investigator’s choice of chemotherapy further proved its superiority over standard chemotherapy and was approved by FDA to treat adult patients with relapsed/refractory B-cell precursor acute lymphoblastic leukemia in August 2017.

## 5. Future Directions

**Figure 6:**
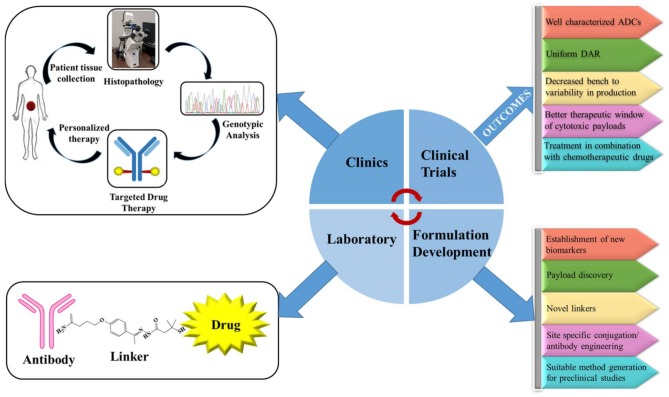
Schematic diagram showing transition of ADCs from laboratory to clinic.

Conventional chemotherapy accounted for consequential toxicities and low therapeutic window for the treatment of malignancies. In the era of personalized medicines, pharmacogenetic testing of the patients followed by ADC treatment can be an excellent alternative over the conventional chemotherapies (Figure 6). For patient selection, a threshold expression of the target antigen must be defined during preclinical development. ADCs also serve as a target-guided tool for the delivery for highly potent cytotoxic drug(s) that cannot be administered as a monotherapy. 

A considerable rise in this field has been observed following the success and FDA approval for Adcetris^®^ in 2011, Kadcyla^®^ in 2013, Besponsa^™^ in 2017 and reapproval of Mylotarg^™^. These recent successes have bolstered ADC developments and presently ~50 ADCs are in pipeline for the treatment of hematologic and solid tumor malignancies. The choice of target, mAb isotype, the linker, the conjugation site and the cytotoxic payload plays crucial part in ADC design. Better understanding of all ADC components may lead to successful generation of an effective ADC. Conventionally, ADC employs a heavily cytotoxic drug as payload (such as calicheamicins, duocarmycins, auristatins, and maytansinoids) however, site specific conjugated ADCs like milatuzumab-Dox, IMMU132, IMMU-130 with moderately cytotoxic payloads like doxorubicin, camptothecin analog SN-38 were also found to be promising, thus redefining the conventional ADC concept. The main challenge remains to optimize the bio-conjugation process to produce homogenous antibody drug conjugates. A better understanding of the role of linker and method of conjugation to the clinical profile of the ADC have led to development of several state of art site-specific conjugation methods for homogenous antibody conjugate production. Table 3 incorporates noteworthy overview(s) on ADCs development through review articles.

Site conjugation processes like Thiomab^®^, enzymatic conjugation, incorporation of unnatural amino acid (unAA) has been used to install reactive handle on the mAb for facilitating a homogenous conjugation process without disrupting the mAb functions. The most common mechanism reported regarding resistances of ADC therapy is attributed to the MDR protein. However, this problem is countered with replacing P-gp substrate drugs with several new naturally occurring toxins, ADC prodrugs as well structural altercations in the drug-linker [38]. A significant effort is also directed towards developing suitable preclinical model to evaluate ADCs therapeutic efficacy. Xenograft bearing mice models does not replicate human conditions genetically in proper way, but genetically engineered mice models are more reliable for evaluating ADCs as they can bear relevant target oncogenes. Another important challenge is to produce cost effective and affordable ADC medications. At present ADCs are quite expansive, for example yearly brentuximab vedotin treatment regimen costs ~$100,000 [125]. In our review, we have put together up-to-date advances in the field of payload discovery, their mechanism of action as well as linker and conjugation technologies. However, regardless of different challenges, recent success in this field can shift the paradigm of cancer therapy to personalized ADCs treatments. 

## Figures and Tables

**Table 1:** Comparison between different side chain conjugation methods.

Conjugation	Reactive Groups	Advantages
Cysteine Residues	Maleimides, haloacetyls, other Michael acceptors	Simple and reproducible method Used in FDA approved Adcetris, widely employed in pipeline candidates, DAR ~0–8 Comparatively less heterogeneous by products than lysine conjugation Easier to characterize pharmacokinetically
Lysine Residues	Activated ester functional groups like *N*-hydroxysuccinimide esters	Though highly heterogeneous, this method is employed in FDA approved Kadcyla^®^, Mylotarg^™^, DAR ~3.5 (Kadcyla^®^), ~2.5 (Mylotarg^™^) Mostly used to crosslink via non-reducible linkers.

**Table 2:** Comparison between different site-specific conjugation technologies.

Method of Conjugation	Reactive Groups	Advantages	Developer
Engineered side chain cysteine residues (ThioMAb) [93]	Maleimides	Improved clinical safety, tolerability and therapeutic index over conventional conjugates. Controlled and reproducible DAR 2. Compatible for producing in large scale.	Genentech
Incorporation of un-natural amino acids (unAA) [94]	Alkoxy-amine	Highly stable and extended half-life in systemic circulation. Improved pharmacological profile compared to conventional ADCs. Ketone group present in unAA provided conjugation site for different alternative payloads like kinase inhibitors, proteasome inhibitors.	Ambrx
Enzymatic Site-Specific Conjugation Process [95]	Amine, Indole	DAR 2-4, More stable conjugates than yielded by ThioMAb and oxime ligation. Controlled conjugation site of the payload on the mAb. Better pharmacokinetic profile over conventional conjugates.	Innate Pharma, Glycos, Pfizer. Inc.

**Table 3:** List of some of the key review articles on ADCs.

Name of the Review Article	Focus of the Review	Year of Publication
Antibody-Drug Conjugates for Cancer Therapy [7]	This article is focused on different key issues like choosing an appropriate target, expression of the target, selecting right mAb isotype.	2008
Antibody Conjugate Therapeutics: Challenges and Potential [122]	The key consideration behind choosing an appropriate target for ADC developments.	2011
Pharmacokinetic Considerations for Antibody Drug Conjugates [10]	Different pharmacokinetic considerations to characterize ADCs as well as PK-PD modellings for development of ADCs	2012
Site-Specific Antibody−Drug Conjugates: The Nexus of Biorthogonal Chemistry, Protein Engineering, and Drug Development [75]	Focuses on methods to synthesize site-specific homogenous ADCs with details of bio-orthogonal chemistries.	2014
Antibody-Drug Conjugates: Design, Formulation and Physicochemical Stability [123]	Physiochemical characterization, formulation considerations, and factors involved in process control.	2015
Methods to Design and Synthesize Antibody-Drug Conjugates (ADCs) [98]	Accounts for different conjugation methods and the chemistry behind in the field of ADCs.	2016
Mechanisms of Resistance to Antibody–Drug Conjugates [35]	Resistance of various ADCs and possible mechanism.	2016
Antibodies and associates: Partners in targeted drug delivery [124]	Engineering antibodies and their subsequent use in different targeted drug delivery systems.	2017
